# Two Liberibacter Proteins Combine to Suppress Critical Innate Immune Defenses in Citrus

**DOI:** 10.3389/fpls.2022.869178

**Published:** 2022-05-02

**Authors:** Supratim Basu, Loan Huynh, Shujian Zhang, Roel Rabara, Hau Nguyen, Jeanette Velásquez Guzmán, Guixia Hao, Godfrey Miles, Qingchun Shi, Ed Stover, Goutam Gupta

**Affiliations:** ^1^Biolab, New Mexico Consortium, Los Alamos, NM, United States; ^2^Horticulture and Breeding, U. S. Horticultural Research Laboratory, Fort Pierce, FL, United States

**Keywords:** *C*Las protein, citrus immunity, HLB pathogenesis, ROS, PCD, bactericidal activity

## Abstract

We adopted a systems-based approach to determine the role of two *Candidatus* Liberibacter asiaticus (*C*Las) proteins, *LasP*_*235*_ and Effector 3, in Huanglongbing (HLB) pathogenesis. While a published work suggests the involvement of these *C*Las proteins HLB pathogenesis, the exact structure-based mechanism of their action has not been elucidated. We conducted the following experiments to determine the structure-based mechanisms of action. First, we immunoprecipitated the interacting citrus protein partners of *LasP*_235_ and Effector 3 from the healthy and *C*Las-infected Hamlin extracts and identified them by Liquid Chromatography with tandem mass spectrometry (LC–MS/MS). Second, we performed a split green fluorescent protein (GFP) assay in tobacco to validate that the interactions observed *in vitro* are also retained *in planta*. The notable *in planta* citrus targets of *LasP*_*235*_ and Effector 3 include citrus innate immune proteins. Third, *in vitro* and *in planta* studies were performed to show that *LasP*_*235*_ and Effector 3 interact with and inhibit the functions of multiple citrus proteins belonging to the innate immune pathways. These inhibitory interactions led to a high level of reactive oxygen species, blocking of bactericidal lipid transfer protein (LTP), and induction of premature programed cell death (PCD), all of which are beneficial to *C*Las lifecycle and HLB pathogenesis. Finally, we performed molecular dynamics simulations to visualize the interactions of *LasP*_*235*_ and Effector 3, respectively, with LTP and Kunitz protease inhibitor. This led to the design of an LTP mimic, which sequestered and blocked *LasP*_*235*_and rescued the bactericidal activity of LTP thereby proving that *LasP*_*235*_, indeed, participates in HLB pathogenesis.

## Introduction

Huanglongbing (HLB) is the most devastating citrus disease (Da Graça et al., 2016; Merfa et al., 2019; Gupta and Stover, 2022). The *Candidatus* Liberibacter asiaticus (*C*Las) colonizes the phloem sieve elements on getting transmitted to the plants during the sap feeding by Asian citrus psyllid (ACP). The *C*Las infection in citrus plants leads mottling of leaves and premature fruit drop. Three α-proteobacteria species (*C*Las, *Candidatus* L. americanus (*C*Lam), and *Candidatus* L. africanus (*C*Laf) are associated with HLB (Prasad et al., 2016). Of these species, *C*Las is the most predominant and virulent species in the USA and vectored by two psyllid species, *Diaphorina citri* Kuwayama and *Trioza erytreae* (Wang and Trivedi, 2013). While endemic in Asia for over a century (Gottwald, 2010; Luo and Agnarsson, 2018), HLB was first encountered about 15 years ago in Florida. Since then, HLB has been widespread in Florida and is looming large on California and Texas, the two other citrus producing states in the USA.

The gram-negative bacteria secrete effector proteins that play an essential role in disease pathogenesis by suppressing multiple proteins belonging to the innate immune system in plants and thereby providing a niche for bacterial colonization and spread in the host (Dodds and Rathjen, 2010). Typically, these effectors are directly injected into the host by the type III secretion system (Feng and Zhou, 2012). The *C*Las is devoid of the type III secretion system (Mudgett, 2005; Feng and Zhou, 2012) but may alternately use type II secretion system to release potential virulence factors or effectors (Sugio et al., 2011; Solé et al., 2015; Cianciotto and White, 2017). These proteins can be encoded by the *C*Las genome or the prophage. The prophages have been shown to exert influence in bacterial pathogenicity as have been seen in *Staphylococcus aureus* (Bae et al., 2006; Zhang et al., 2011). Two autotransporter proteins (LasA_I_ and LasA_II_) with leucine-rich repeats (LRRs) have been identified in Las psy62 prophage regions and have been shown to target the mitochondria in plants (Hao et al., 2013). Note that *C*Las codes for a smaller number of effectors because of the small (1 Mb) genome-size (Duan et al., 2009; Lin et al., 2013). The gram-negative bacteria with 5 Mb genomes have several 100 unique effectors (Dillon et al., 2019) as opposed to about 80 effectors identified, so far, from the *C*Las genome (Pitino et al., 2016, 2018; Prasad et al., 2016). However, the interactome studies revealed the ability of a single effector to bind multiple protein from the host plant (Block et al., 2008; Büttner, 2016). Thus, the bacteria have evolved to utilize a smaller number of effectors to establish a niche for pathogenesis in the host. Therefore, it was of importance whether to determine the two *C*Las proteins, *LasP*_*235*_ and Effector 3, may target multiple citrus proteins, suppress immunity, and contribute to HLB pathogenesis.

In this study, we focused first on identifying the critical steps associated with the breakdown of citrus innate immune defense in response by the *C*Las effectors. Typically, the plant innate immune defense involves multiple pathways including pathogen or microbe-associated molecular pattern (MAMP)-triggered immunity (MTI); pattern-triggered immunity (PTI), effector triggered immunity (ETI), and plant hormone, such as salicylic acid (SA), jasmonic acid (JA), and ethylene (ET), induced immunity (He et al., 2007; Wu L. et al., 2014; Brauer et al., 2018; Qi et al., 2018; Zhang et al., 2018; Alhoraibi et al., 2019). The PTI or MTI provides the first line of plant defense against pathogens or microbes through the recognition of PAMP or MAMP, such as bacterial liposaccharide (LPS), elongation factor thermal unstable (EF–Tu), flagellin. The PAMP or MAMP recognition is mediated by the plasma membrane pattern recognition receptors (PRR) that include LRR, flagellin receptor (FLS2), and EF–Tu receptor (EFR). The plasma membrane PRR recognition induces intracellular mitogen-associated protein kinase (MAPK) signaling leading to the expression of pathogen-related (PR) or defense genes (Dangl and Jones, 2001; Sels et al., 2008; Ali et al., 2018). However, the pathogen effectors can block both intracellular and extracellular steps in the PTI pathway (Cui et al., 2009; Büttner, 2016). To counter the pathogen induced blocking of the PTI pathway, the plants have evolved the ETI pathway in which the intracellular nod-like receptors (NLR) recognize the pathogen effectors and augment the MAPK signaling and PR gene expression. The ETI pathway also induces hypersensitive response through the production of reactive oxygen species (ROS), which causes cell death at the site of the infection thereby limiting the pathogen spread. The PTI and ETI pathways also couple to intracellular plant hormone SA/JA/ET pathways, which also involve ROS production and induction of PR genes. It has been demonstrated that the effectors from plant pathogenic bacteria can inhibit one or more steps in these pathways (Medina et al., 2018; Mine et al., 2018; Han and Kahmann, 2019; Lee et al., 2019). Also, the bacterial effectors are known to subvert multiple steps leading to programed cell death (PCD) in plant, which is a form of immune defense by PTI and/or ETI to control infection (Hoeberichts and Woltering, 2003; Abramovitch and Martin, 2005; Locato and De Gara, 2018). Therefore, it was of interest for us to determine which steps in the citrus innate immune defense are affected by the proteins encoded by the *C*Las genome and prophage.

First, we performed *in vitro* and *in planta* studies to identify the prominent citrus proteins targeted by *LasP*_*235*_ and Effector 3. Second, we performed functional assays to determine whether *LasP*_*235*_ and Effector 3 have inhibitory effects on their citrus protein targets. Third, we performed molecular dynamic simulations to analyze the details of interaction between *LasP*_*235*_ (and Effector 3) and their selected citrus targets and predicted which pairwise interactions are critical for inhibition of the citrus target function. Finally, we validated our prediction of the inhibitory mechanism by site-specific mutations on the citrus protein(s) that affect the critical pairwise interactions. We discovered that each of the two effectors can directly target several citrus innate immune proteins. A clear understanding of the inhibitory mechanisms will provide guidelines for countering *C*Las effectors and developing anti-infectives to block HLB pathogenesis.

## Materials and Methods

### Experimental Procedures

#### Plant Materials and Growth Conditions

Hamlin trees verified as being HLB-free and ACP-friendly were purchased and placed in the green house. One branch cage placed in the upper part of each tree (three replicates) was filled with 75ACP from an infected population while other trees had cages with clean 75ACPs placed serving as control. The insects were allowed to feed on the trees for a week and then the insects were killed by spraying with topical insecticide. The ACPs were tested for *C*Las and the trees were subsequently returned to the greenhouse. The leaf samples (12 for each biological replicate and treatment) were collected from the untreated and infected plants and flash-frozen in liquid nitrogen and stored for further analysis.

#### Cloning and Overexpression of Effectors and Targets in *Escherichia coli*

The genes from *Liberibacter asiaticus* (non-culturable bacteria) were identified (*LasP*_*235*_ and Effector 3), codon optimized and cloned in pUC57 by GenScript. The effectors were then amplified and cloned in pET28(a) vector between NdeI and BamHI sites and transformed in *E. coli* BL21 [BL21(DE3)pLysS]. The positive clones were inoculated overnight in LB with Kanamycin. The overnight culture (1%) was grown until the Optical density (OD) reached 0.6 and then induced with IPTG at 30°C for overnight. The cells were harvested next day and resuspended in protein isolation buffer (20-mM Tris-Cl, pH 7.4, 150-mM NaCl, and 10% glycerol). The cell suspension was sonicated and centrifuged at 14,000 rpm, 4°C, 30 min. The supernatant was collected and the inclusion bodies were treated with 9M urea. Following the treatment with urea, the cell suspension was centrifuged and supernatant was collected and refolded. The refolded protein from the inclusion bodies and the soluble fractions were purified using TALON metal affinity resin (Joshi and Puri, 2005).

#### Isolation of Total Protein From Citrus

Fresh leaf tissue, from five Hamlin trees (*Citrus sinensis* L. Osbeck) was pulverized in liquid nitrogen using a pestle and mortar and the resulting fine powder stirred with 1.5 volumes of extraction buffer [50-mM HEPES pH 7.5, 5-mM EDTA, 5-mM EGTA, 10-mM dithiothreitol (DTT), 10% glycerol, 7.5% polyvinylpolypyrolidone (PVPP), and a protease inhibitor cocktail, Complete™, Boehringer Mannheim]. The slurry was subsequently mixed on a reciprocating shaker (100 oscillations per min) for 10 min, at 4°C, followed by centrifugation 15,000 *g* for 30 min at 4°C. The supernatant was removed and immediately flash-frozen in liquid nitrogen and stored at −80°C until needed (Roy et al., 2011).

#### Pull Down Assay and LC–MS/MS Analysis to Identify Citrus Targets

The purified refolded effector proteins were incubated with total protein (15 μg) isolated from healthy and infected citrus leaf extract for 2 h at 4°C. The effector–protein complex was incubated with TALON metal affinity resin at 4°C overnight. The resin was washed with column buffer (50-mM Tris-Cl, pH 7.4, 150 mM, 10% glycerol) and eluted with imidazole (250 mM). The eluted protein complex was sent for LC–MS/MS analysis to identify the citrus targets (Zhang et al., 2017). The spectra were searched against the Uniprot database, and taxonomy was set to *C. sinensis*. The only peptides that were ranked 1 were selected and finally those targets were selected for further analysis that had a 95% confidence (Karpievitch et al., 2012).

### Enzymatic Assays and Their Inhibitions by the *C*Las Effectors

#### Superoxide Dismutase Assay

The superoxide dismutase (SOD) assay was quantified based on its ability to inhibit the photochemical reduction of nitroblue tetrazolium (NBT) by superoxide radical and assayed following (SOD Kit; catalog No.: 7,500-100-K) with some modifications. The reaction mixture (3 ml) contained 13 mM methionine, 75-mM NBT, 2-mM riboflavin,100-mM EDTA, and 0.3-ml leaf extracts. The volume was made up to 3 ml using 50-mM phosphate buffer with the addition of riboflavin at the very end. Once the reaction mixture was made, they were mixed well and incubated below two 15-W fluorescent tubes with a photon flux density of around 40 mmol m^−2^ s^−1^ for 10 min. Once the reaction is completed, the tubes were covered with a black cloth and the absorbance was measured at 560 nm. The non-irradiated mixture served as control and the absorbance so measured is inversely proportional to the amount of enzyme added. The SOD activity is defined as the amount of enzyme that caused 50% inhibition of the enzymatic reaction in the absence of the enzyme (Basu et al., 2010). The presented data were an average of three biological replicates, and two of them were of technical replicates.

#### Aspartyl Protease Assay

The protease assay was performed using a fluorescence based (BODiPY) EnzChek protease assay kit. The analysis of aspartyl protease activity was done by incubating it with no other proteins in sodium citrate buffer (50 mm, pH 4.5). To perform the inhibitory effect of *LasP*_*235*_ on the protease activity the renatured aspartyl protease was preincubated with increasing concentrations of *LasP*_*235*_ at 4°C for 2 h in sodium citrate buffer. Following incubation, BODiPY-labeled casein substrate was added, and the reaction was monitored by measuring fluorescence in Tecan Infinite 200 PRO microplate reader at 485 ± 12.5 nm excitation/530 ± 15 nm emission filter. The assays were conducted in triplicates (Leippe et al., 2011; Coria et al., 2016).

#### Glycosyl Hydrolase Assay

The inhibitory effect of recombinant *LasP*_*235*_ on recombinant glycosyl hydrolase was assayed using the β-Glucosidase Activity Assay Kit (MAK129, Sigma). The enzymatic reactions were carried out in K-Phosphate buffer (100 mM, pH 6.5) with *p*-nitrophenyl-β-D-glucopyranoside (β-NPG) for 20 min at 37°C. The final absorbance of the hydrolyzed product was measured at 405 nm (Henriques et al., 2017).

#### Aldehyde Dehydrogenase Assay

This assay was performed using aldehyde dehydrogenase (ALDH) Activity Abcam Assay Kit with modifications. In short, the purified ALDH was incubated with increasing concentration of substrate (acetaldehyde) for 1 h. The absorbance was measured at 450 nm and expressed in terms of NADH standard as mU/ml (Ouyang et al., 2019). The presented data were an average of three biological replicates, and two of them were of technical replicates.

#### Trypsin Inhibition Assay

The trypsin inhibition assay was done in triplicate and the result was expressed as a means of three replicates. In short, the residual trypsin activity was measured by monitoring the change in absorbance at 247 nm in presence of increasing concentration of recombinant purified Kunitz Trypsin inhibitor (KTI) when incubated with *p*-toluene-sulfonyl-l-Arg methyl ester (Sigma; Nehir El et al., 2015). The presented data were an average of three biological replicates, and two of them were of technical replicates.

#### *In planta* Split GFP Assay (Agro-Infiltration)

*Agrobacterium tumefaciens* LBA4404 transformant cells carrying *Las*_*P*235_, Effector 3 and the targets from citrus plants (Aspartyl protease, glycosyl hydrolase, SOD, KTI protein, lectin etc.), respectively, are cloned in pR101 vector and cultured overnight in LB medium with 50 μg ml^−1^ of rifampicin and 50 μg ml^−1^ kanamycin, and resuspended in 10-mM MgCl_2_, 10-mM MES. The culture was diluted to an optical density of 0.5 (OD 600 nm). For each effector–target interaction, three leaves of 4 *Nicotiana benthamiana* plants overexpressing GFP1-9 were infiltrated with the *A. tumefaciens* suspension containing the effector and the target plasmids, respectively. The agro-infiltrated leaves were analyzed for protein localization at 3 dpi under a microscope (Olympus BX51-P) equipped with a UV light source. The agroinfiltrated plants were kept in a greenhouse for 24 h and the interaction was visualized using Illumatool lighting system (LT-9500; Lightools Research) with a 488-nm excitation filter (blue) and a colored glass 520-nm long pass filter. The photographs were taken by Photometric CoolSNAP HQ camera (Cabantous and Waldo, 2006; Liu et al., 2018).

#### Estimation of Superoxide Anion

The leaf disks from agro-infiltrated tobacco plants were incubated at 25°C on a shaker for 30 min in dark in 1 ml of K-phosphate buffer (20 mM, pH 6.0) containing 500-μM XTT. The increase in absorbance was measured at 470 nm in a spectrophotometer (Ramegowda et al., 2012). The presented data were an average of three biological replicates, and two of them were of technical replicates.

#### Lipid Binding and MIC Assays for Lipid Transfer Protein

The lipid binding activity of recombinant LTP-6X His protein overexpressed and purified from *E. coli* was mixed with of 2-*p*-toluidinonaphthalene-6-sulphonate (TNS) at 25°C. The results were recorded at excitation 320 nm and the emission at 437 nm. The inhibitory action of *LasP*_*235*_ on lipid transfer protein (LTP) was assessed using increasing concentration of *LasP*_*235*_ and the results were measured. The purified GFP was used as a control (Melnikova et al., 2020). The minimum inhibitory concentration (MIC) of the LTP was performed using broth microdilution technique. The assay was carried out using 5 × 10^5^ colony forming units (CFU ml^−1^) in MHB. The MIC was defined as the lowest concentration of the protein required to inhibit the visible growth of bacterial strains used (Ebbensgaard et al., 2015). The presented data were an average of three replicates, and two of them were of technical replicates.

#### Estimation of Ion Leakage From Leaf Disks

*Agrobacterium tumefaciens* LBA4404 transformant cells carrying Effector 3 and the targets from citrus plants KTI protein cloned in pR101 vector was cultured overnight in LB medium with 50 μg ml^−1^ of rifampicin and 50 μg ml^−1^ kanamycin and resuspended in 10-mM MgCl_2_, 10-mM MES. The culture was diluted to an optical density of 0.5 (OD 600 nm). For the assay, three leaves of N. *benthamiana* plants previously treated with paraquat (PQ; 100 μM) were infiltrated with the *A. tumefaciens* suspension containing the effector alone, Kunitz alone and the mixture of effector 3 and Kunitz, respectively (Pitino et al., 2016, 2018) and incubated for 48 h. The leaf disks were prepared by punching the leaf disks with a cork puncher. The punctured leaf disks were placed in water (50 ml) for 5 min to mitigate the error of measuring ion leakage due to injury inflicted on the leaves due to puncturing. The water was removed and the leaf discs were incubated with 5 ml. the conductivity was measured after 3 h using Mi180 bench meter and this value is referred to as *A*. The leaf disks with the bathing solution were then incubated at 95°C for 25 min and then cooled to room temperature to enable complete ion leakage. The conductivity was measured again, and this value is referred to as *B*. The ion leakage is subsequently expressed as (*A/B*) × 100. All experiments were carried out in three biological replicates with five leaf disks for each sample (Wu et al., 2017; Hatsugai et al., 2018).

### Pathogen Inoculation and LTP Treatment in *N. benthamiana* Leaves

*Pseudomonas syringae* pv. *Tomato* DC3000 was cultured on King'S B (KB) medium containing 50 μg ml^−1^ rifampicin. Overnight, log-phase cultures were grown to an optical density at OD_600nm_ of 0.6–0.8 (OD 0.1 = 10^8^ cfu ml^−1^) and diluted with 10-mM MgCl_2_ to the concentrations of 10^5^ CFU ml^−1^ before inoculation. Control was performed with 10-mM MgCl_2_. The bacterial suspensions were infiltrated into the abaxial surface of a leaf using a 1-ml syringe without a needle. *Agrobacterium tumefaciens* LBA4404 transformant cells carrying *LasP*_*235*_ and LTP protein cloned in pR101 vector was cultured overnight in LB medium with 50 μg ml^−1^ of rifampicin and 50 μg ml^−1^ kanamycin and resuspended in 10-mM MgCl_2_, 10-mM MES. The culture was diluted to an optical density of 0.5 (OD 600 nm). For the assay, the infected leaves of *N. benthamiana* plants were infiltrated with the *A. tumefaciens* suspension containing the LTP alone, LTP+ *LasP*_*235*_, different mimics (Liu et al., 2013). The presented data were an average of three replicates, and two of them were of technical replicates.

### The qPCR Analysis

The concentration, quality, and integrity of the DNA was analyzed using the Agilent 2100 bioanalyzer (Bio–Rad) and NanoDrop™ ND-1000 (Thermo Scientific). The qRT-PCR experiments were conducted using GoTaq qPCR Master Mix (Promega), *P.syringae* gene-specific primers (Psy-F ATGATCGGAGCGGACAAG; Psy-R GCT CTT GAG GCA AGC ACT), and *PR1b* (pathogenesis related protein): PR1F-CGAGAGGCCAAGCTATAACTAC and PR1R: GCAAGAAATGAACCACCATCC gene from tomato genome was used a as standard to show that the variation in Ct values was due to infection and treatment with LTP or the mimics (Guilbaud et al., 2016). The experiments were carried out with three biological replicates and each replicate divided into two technical replicates in a CFX-96 Bio–Rad thermocycler (Bio–Rad). Increasing temperature (0.5°C 10 s^−1^) from 55 to 95°C was used for melt curve analysis. The bacterial load corresponding to the CFU was calculated from standard curve. To determine the sensitivity of the qPCR assay, a culture of bacterial strain was diluted with sterile water to generate a 10-fold dilution series from 1 × 10^8^ to 1 × 10 CFU·ml^−1^. Each dilution (1 μl) of bacterial suspension was used as templates for quantifying bacterial load by direct qPCR without a DNA extraction step. The resulted Ct values were plotted against the corresponding CFU·ml–^1^ value to generate a standard curve for the detection limit. Each dilution was analyzed in three replicates.

### Data Analysis

For each of the investigated parameters, the experiments were conducted in triplicate with two technical replicates. All experimental data values were expressed as means of three measurements [± standard error (SE)]. The significance of the differences between the mean values were statistically evaluated by two-sided *t*-test at *p* ≤ 0.05 using the Windows 2004/Microsoft Excel computer package for significance. The *K*_m_ (Michaelis constant), *V*_max_ were calculated using Lineweaver Burk plot. The *K*_cat_ = *V*_max_/[*E*], where *E* is the total enzyme, i.e., free enzyme and enzyme bound to the substrate. IC_50_ = (Concentration of the effector protein × 50)/% inhibition.

### Molecular Modeling

#### Prediction of Protein 3D Structures and Complexes

The 3D structures of the two *CLas* proteins (*LasP*_*235*_ and Effector 3) and the two citrus proteins (LTP and KTI) were predicted using I-TASSER (https://zhanglab.ccmb.med.umich.edu/I-TASSER/). We then used HADDOCK version 2.2 webserver to predict interaction interfaces of *LasP*_*235*_-LTP and Kunitz-E3 complexes (http://milou.science.uu.nl/services/HADDOCK2.2/). The selected complexes of *LasP*_*235*_-LTP and Kunitz-E3 were further refined using molecular dynamics (MD) simulations of these complexes in the presence of water.

### Protein-Water System Setup for MD Simulation

Our simulations started with single protein (i.e., LTP, *LasP*_*235*_, Kunitz or E3) in water. These systems contained 10,000 water molecules in a box of 6.9 × 6.8 × 7.1 nm. To refine the models of *LasP*_*235*_-LTP and Kunitz-E3 obtained from HADDOCK. We conducted MD simulations of these complexes in the presence of water. The protein–protein complex systems contain 30,000 water molecules in a box of 9.9 × 9.9 × 9.9 nm with excess NaCl at 150 mM to mimic experimental conditions. For Kunitz-E3 complexes, we focus on model with Kunitz's active loop in close contact with E3's interface that contain either aspartic acid or glutamic acid residues or a large hydrophobic surface. For *LasP*_*235*_-LTP complexes, we focus on model with LTP's lipid entrance site B1 and B2 (see Supplementary Figure 3) in close contact with *LasP*_*235*_. Following the MD simulation, the systems with stable complexes and adequate protein–protein pairwise residues interactions were then further validated by extended MD simulation.

### Protein–Bilayer System Setup for MD Simulation

Our simulations started with a single LTP in the water and a mimetic of the *E. coli* inner membrane composed of a 3:1 ratio of 1-palmitoyl-2-oleoyl-*sn*-glycero-3-phosphoethanolamine (Wu et al., 2017) (POPE) and 1-palmitoyl-2-oleoyl-*sn*-glycero-3-phosphoglycerol (POPG). The lipid bilayers are constructed with the Charm–GUI membrane builder (Wu E. L. et al., 2014) followed by 40 ns of *NpT* simulation at 310 K with semi-isotropic pressure coupling. The LTP–bilayer system contained 10,000 water molecules and 128 lipid molecules in a box of 6.1 × 6.1 × 12.5 nm. We also conducted simulation of *LasP*_*235*_-LTP complex in the bilayer POPE: POPG (3:1 ratio) to further refine the *LasP*_*235*_-LTP models obtained from MD simulation of the *LasP*_*235*_-LTP complexes in the water. The *LasP*_*235*_-LTP/bilayer contained 23,600 water molecules and 256 lipid molecules in a box of 8.7 × 8.7 × 13.7 nm. The LTP, or *LasP*_*235*_-LTP complex, was placed 3.5 nm away from the center of mass of the lipid bilayer along its normal. The protein/bilayer systems were neutralized and excess NaCl was added at 150 mM to mimic experimental conditions.

#### Simulation Protocol

For the MD simulations, the TIP3P water model was used with CHARMM modifications (Albaugh et al., 2016). The water molecules were rigidified with SETTLE (Miyamoto and Kollman, 1993) and the molecular bond-lengths were constrained with P-LINC Lennard–Jones interactions (Wennberg et al., 2013) were evaluated using a group-based cutoff, truncated at 1 nm without a smoothing function. The Coulomb interactions were calculated using the smooth particle–mesh Ewald method (Cerutti et al., 2009; Kratz et al., 2016; Boateng, 2020) with a Fourier grid spacing of 0.12 nm (Fischer et al., 2015). The simulation in the *NpT* ensemble was achieved by semi-isotropic coupling at 1 bar with coupling constants of 4 ps (Aoki and Yonezawa, 1992; Blumer et al., 2020) and temperature-coupling the simulation system using velocity Langevin dynamics with a coupling constant of 1 ps (Washio et al., 2018). The integration time step was 2 fs. The non-bonded pair-list was updated every 20 fs (Walser et al., 2002).

## Results

### *In vitro* Protein Assay to Identify the Citrus Protein Targets of *LasP*_*235*_ and Effector 3

The *LasP*_*235*_ identified in the prophage region of Las genome encoded a 123 amino-acid protein has an N-terminal nuclear localization signal (NLS) with no typical signal peptide (Hao et al., 2019). Effector 3 on the contrary has a predicted chloroplast targeting signal sequence (Pitino et al., 2016, 2018). The homology modeling predicted the presence of helical bundles in the structure of *LasP*_*235*_ as shown in Supplementary Figure 1A. Note that the similar helical bundles are also present in AvrRps4, a *P. syringae* effector involved in plant immunity (Sohn et al., 2012). It is suggested that the helical effectors from bacteria may interact with multiple plant helical proteins *via* intermolecular coiled–coil interactions (Chan et al., 2010; Goritschnig et al., 2016). These proteins may be located on the plasma membrane, in the cytosolic fluid or vacuole, and in the nucleus. The homology modeling also predicted two helix bundles in the structure of Effector 3 in addition to a disordered C-terminal segment (see Supplementary Figure 1B). The latter may make Effector 3 a promiscuous binding partners of several citrus proteins. In addition, due to the presence of chloroplast targeting signal, Effector 3 may be a potential *CLas* effector. Note that multiple chloroplast proteins are involved in ROS production and plant hormone signaling (Sowden et al., 2018), which may mediate cell death as an innate immune defense.

**Figure 1:**
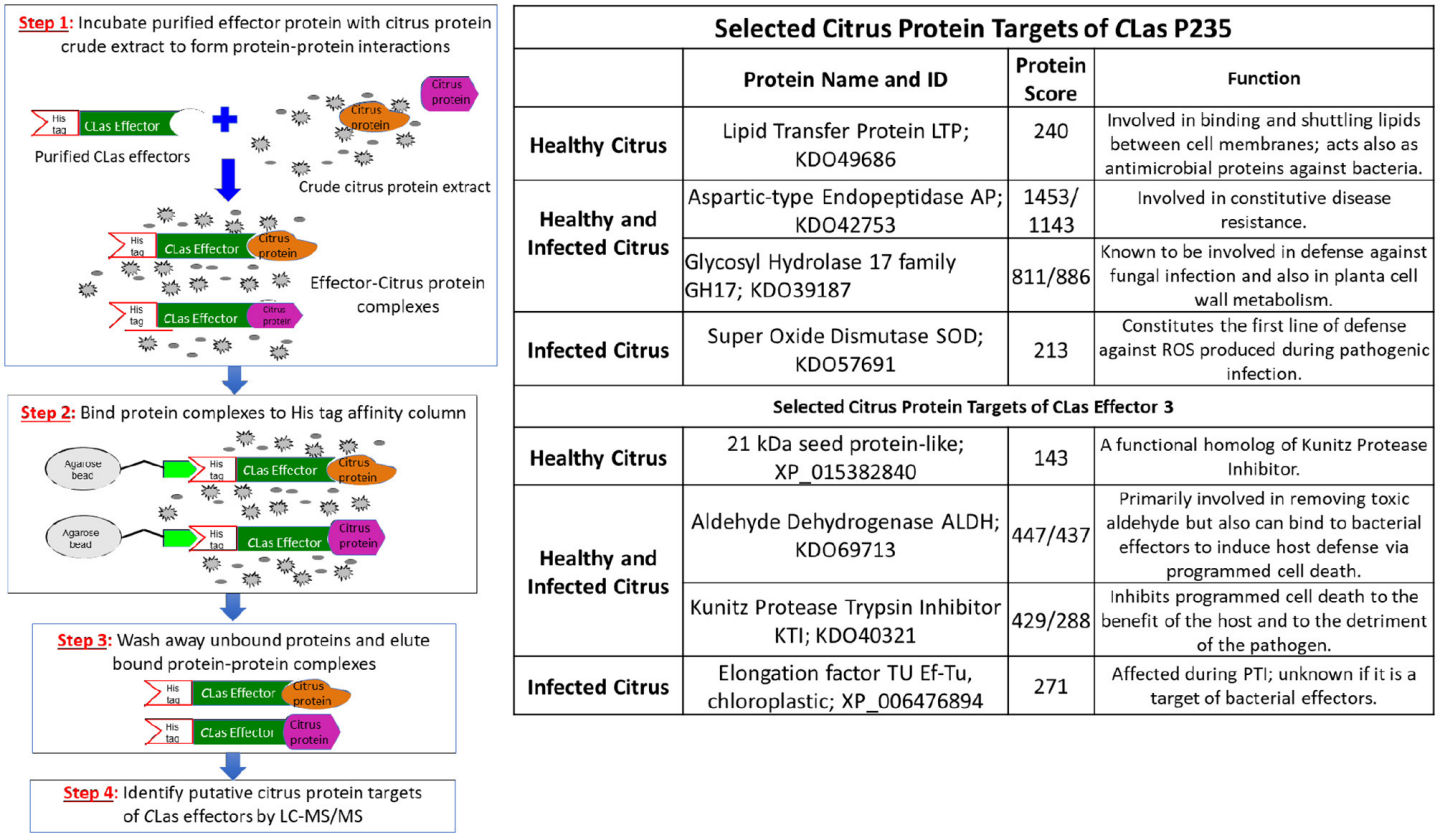
Identification of citrus target proteins of the *C*Las effectors. (Left) Outlines of the experimental steps. Step 1. *C*Las effectors were overexpressed in *E. coli* with His_6_ tag. Purified His_6_-tagged effectors (colored green) were incubated with protein extracts from healthy and infected Hamlin citrus. Specific citrus target protein (colored orange and magenta) bound to the *C*Las effectors. Step 2. Effector-target complexes were TALON on Agarose beads and non-target citrus proteins were washed away. Step 3. The specific effector-target complexes were eluted. Step 4. The citrus target proteins from the eluted complexes were identified by LC–MS/MS. (Right) Selected citrus protein targets of the *C*Las effectors, *LasP*_*235*_ and Effector 3. The citrus targets were chosen on the basis of their high protein scores. The target proteins were selected both from healthy and *C*Las-infected protein extracts. GenBank sequence IDs and putative functions (based on the literature data) of the citrus targets are listed.

The steps in our target identification scheme are shown in Figure 1 (left). First, we expressed *LasP*_*235*_ and Effector 3 in *E. coli* with C-terminal His_6_-tags. The Effector 3 was expressed without the signal sequence. Both proteins were extracted from the inclusion body and re-folded. Second, the His-tagged *LasP*_*235*_ and Effector 3 were bound to TALON columns and were incubated with citrus protein extracts from the uninfected and *CLas*-infected Hamlin. Third, bound citrus protein targets were eluted from the column and identified by LC–MS/MS. Finally, the spectra from LC–MS/MS were searched against the Uniprot database with taxonomy set to *C. sinensis*. The highest ranked citrus proteins, in terms of the LC–MS/MS protein score (Koenig et al., 2008), were selected as putative targets of *LasP*_*235*_ and Effector 3. See Supplementary Tables 1A,B for all citrus targets of *LasP*_*235*_ and Effector 3 with high protein scores. Supplementary Table 1C lists the background targets as obtained by first incubating the TALON column with the protein extraction buffer and then elution by the citrus protein extract. Note that the non-specific targets with low protein scores were obtained by buffer elution. As shown in Figure 1 (right), the top-ranked citrus targets of *LasP*_*235*_ and Effector 3 show protein scores far greater than those listed for the non-specific targets in Supplementary Table 1C. A subset of these targets was further analyzed. The selected *LasP*_*235*_ targets are SOD (from infected citrus), LTP (from healthy citrus), Aspartyl Endopeptidase, (AP) and Glycosyl Hydrolase family 17, GH17 (from both healthy and infected citrus) whereas the Effector 3 targets are as follows: KTI and ALDH from both healthy and infected citrus, Elongation Factor Tu (Ef–Tu) from infected citrus, lectin, and 21 kDa seed protein-like (a functional homolog of KTI) from healthy citrus. As indicated, all target proteins listed in Figure 1 (right) are involved in citrus innate immunity. Although, it allows identification of both extracellular and intracellular targets of *CLas* effectors from infected and healthy citrus, our method in Figure 1 is likely to miss the citrus targets that are expressed at a low level.

### *In planta* Validation of the Citrus Protein Targets of *LasP*_*235*_ and Effector 3

**Figure 2:**
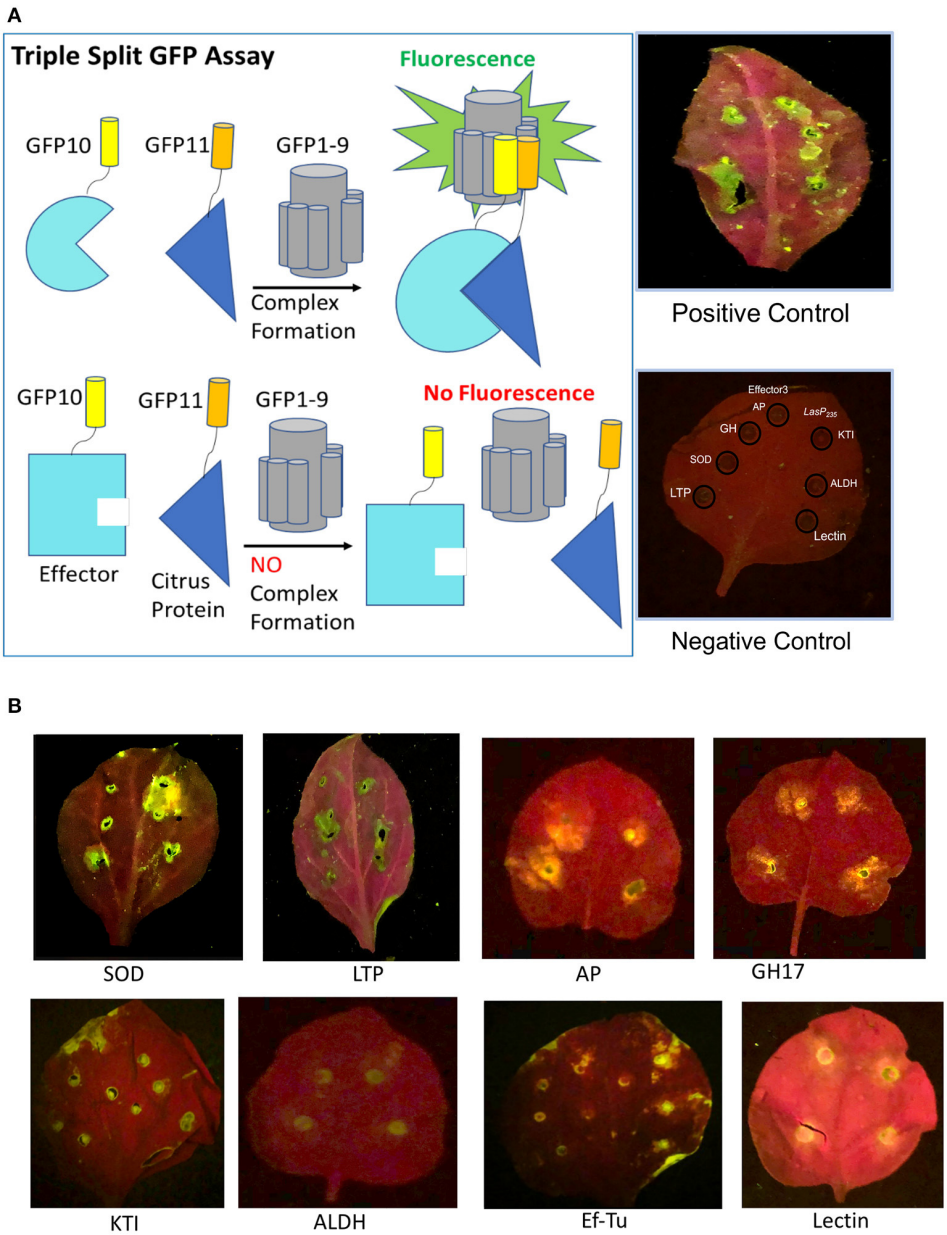
*In planta* validation of the selected citrus protein targets of *LasP*_*235*_ and Effector 3 by triple split GFP assay. [**(A)**, left] A schematic representation of the principle of the triple split GFP assay. Green fluorescence is observed when the Effector (cyan) linked to GFP10 (yellow) interacts with the target (blue) linked to GFP11 (orange) and the effector-target complex complements with GFP1-9. There is no fluorescence in the absence of an interaction. [**(A)**, right] The presence of fluorescence when agrobacterium carrying enhanced GFP is infiltrated on the leaves of transgenic tobacco expressing GFP1-9 is used as a positive control. Absence of fluorescence when the leaves of transgenic tobacco expressing GFP1-9 co-infiltrated with *LasP*_*235*_ and the interactors of Effector 3 in one-half of the leaf (or Effector 3 and the interactors of *LasP*_*235*_ in the other half of the leaf) served as the negative controls. Note the absence of fluorescence. Under the 488 nm excitation filter (blue) and colored glass 520 nm long pass filter, the chlorophyll background appears as red and fluorescence at the site of co-infiltration of the effector and its target appears as greenish yellow spots. **(B)** Complex formation when *LasP*_*235*_ is co-infiltrated with SOD, LTP, AP, and GH17 in the leaves of GFP1-9 transgenic tobacco (top panel) and when Effector 3 is co-infiltrated with Effector 3 and KTI, ALDH, Ef–Tu, and Lectin (bottom panel).

*In planta* validation is based on a split triple GFP assay, which has been successfully applied to monitor protein–protein interactions in yeast, human, and plant (Cabantous et al., 2013; Pedelacq et al., 2019). The assay relies on the principle that specially enhanced 11 stranded GFP can be split into GFP1-9, GFP10, and GFP11 with none of the three split components showing fluorescence. However, the fluorescence is recovered when GFP1-9, GFP10, and GFP11 are re-assembled. We constructed stable transgenic tobacco lines that overexpress GFP1-9 as a detector of *in planta* protein–protein interactions. The two agrobacterium constructs, i.e., one overexpressing *LasP*_*235*_ (or Effector 3) with a GFP11 tag and the other a putative citrus target with a GFP10 tag, were infiltrated in the GFP1-9 transgenic tobacco. As shown in the experimental design of Figure 2A, we expect to observe (i) green fluorescence in the presence of a target–effector interaction and (ii) no fluorescence in the absence of interaction. In our assay, for negative controls (see Figure 2B), we confirmed the lack of interaction between Effector 3 and the targets for *LasP*_*235*_ (and the lack of interaction between *LasP*_*235*_ and the targets for Effector 3). Agrobacterium carrying enhanced GFP was used as a positive control. Note that the leaves in the negative control appear red in color due to chlorophyll autofluorescence. The fluorescence coming from chlorophyll molecules is a result of emission characteristics of both individual chlorophyll molecules and the fluorescence is observed at excitation and emission maxima of 685 and 720–730 nm. Figure 2B (top) shows the results of the split GFP assay monitoring the interaction of *LasP*_*235*_. Note the presence of green fluorescence at the infiltrated leaf sites for SOD, LTP, AP, and GH17, which were identified as putative targets of *LasP*_23_ as identified from our *in vitro* protein assay as described Figure 2A. The pattern of fluorescence is comparable to the infiltration of agrobacterium carrying enhanced GFP. Thus, the split GFP assay shows specific *in planta* interactions between *LasP*_*235*_ and citrus proteins (SOD, LTP, AP, and GH17). Figure 2B (bottom) shows the results of the split-GFP assay monitoring the interaction of Effector 3. The presence fluorescence at the filtrated sites indicates specific *in planta* interactions between Effector 3 and (KTI, ALDH2, lectin, and Ef–Tu) that were identified by the *in vitro* protein assay. Triple split GFP assay provides the following advantages (Serebriiskii et al., 2000; Rajagopala, 2015) over other commonly used assays such as yeast-two hybrid system for monitoring protein–protein interaction: (i) It can be readily adapted to *in planta* systems; (ii) It limits false positives and negatives; (iii) Small GFP10 and GFP11 tags retain native effector–target interactions; (iv) Positive and negative controls can easily be incorporated for *in planta* measurement to improve the fidelity of the assay.

### The Two *C*Las Proteins Inhibit the Functions of Their Specific Citrus Targets

#### *In vitro* Assays

Three targets of *LasP*_*235*_, i.e., SOD, AP, and GH17, that are validated by *in planta* split GFP assay, are citrus PR or defense proteins with enzymatic activities. As described in the “Methods,” the citrus target proteins were expressed in *E. coli*, extracted from the inclusion body, and purified by affinity purification schemes. After purification, the proteins were re-folded. The identity of the proteins was confirmed by western blot analysis (Supplementary Figure 2) and Mass spec analysis (data are not shown). Therefore, before conducting *in vitro* inhibition assays, it was necessary to determine the enzymatic activities of the recombinant enzymes to confirm that they retained the native fold and function. We then determined the inhibitory activity of *LasP*_*235*_ on them by measuring IC_50_ (the concentration required for 50% reduction in enzymatic activity). The SOD, which is unique to plants, prevents damage caused by the ROS burst upon pathogen infection (Miller, 2012). While it facilitates the direct killing of the pathogen and induction of plant defense genes, excessive ROS is damaging to the plant. The SOD, produced in mitochondrion, peroxisome, and chloroplast, converts oxygen radical to molecular oxygen and hydrogen peroxide. The latter, also potentially phytotoxic, is subsequently converted by plant catalase into molecular oxygen and water. The SOD is also involved in regulating ROS signaling leading to the induction of defense genes (Wang et al., 2018). As shown in Table 1, *LasP*_*235*_ inhibits the activity of the citrus SOD. The Citrus AP belongs to the A1 family of atypical aspartate proteases, primarily located in apoplast and chloroplast. It has been shown that an atypical aspartate protease, expressed in the apoplast, confers constitutive disease resistance 1 (CDR1) in *Arabidopsis* probably by producing a peptide ligand through cleavage and subsequent induction of SA signaling and expression of PR genes (Varghese et al., 1994; Simões et al., 2007). The results of enzyme assay show that *LasP*_*235*_ inhibits the activity of the citrus AP. The GH17, a citrus (β1-3) glucanase, is another direct interactor of *LasP*_*235*_. Typically, GH17 glucanases are known to provide disease resistance against fungi by hydrolyzing fungal chitins (Hrmova and Fincher, 2001). However, GH17 also has a role in immune defense in general in that it regulates the formation of callose (a β1-3 glucan polysaccharide), which is an essential component of papillae, an ultrastructure formed at the site of pathogen penetration. Apart from callose, the papillae also contain ROS and antimicrobial peptide thionin and thus provide the first line of defense against pathogen invasion. In papillae-mediated immunity, callose may be involved in two different mechanisms of plant defense against pathogens. First, the callose deposition in papillae may block pathogen spread. Second, the hydrolyzed products of callose by GH17 may serve as ligands for the PRRs and may induce SA signaling leading to plant immune defense. Thus, the GH17-mediated hydrolysis of callose may either support pathogen spread or induce SA signaling. As evident from Table 1, *LasP*_*235*_ inhibits the glucanase activity of the citrus GH17. Therefore, it may induce SA signaling and help *C*L*as* to suppress citrus immune defense. The citrus LTP is the non-enzyme direct interactor of *LasP*_*235*_. The plant LTPs possess (i) lipid binding property, which is critical to lipid homeostasis and membrane dynamics and (ii) bactericidal activity as a component of immune defense (Liu et al., 2015; Finkina et al., 2016; Salminen et al., 2016; Shenkarev et al., 2017). Table 1 shows that *LasP*_*235*_ can block both lipid-binding and antimicrobial activities. Table 1 shows inhibitory activities of Effector 3 on two citrus target proteins: ALDH, which converts aldehydes into carboxylic acid using NADPH/NADH as a co-factor (Jimenez-Lopez et al., 2016) and KTI, which inhibits protease activity of PCD-inducing trypsin (Li et al., 2008).

**Table 1:** The citrus AP, SOD, GH17, and ALDH were overexpressed in and purified from *E. coli* and enzymatic assays were performed on them following the protocols described in Experimental Procedures section.

	**Citrus targets**	**K_**cat**_ (s^**−1**^)**	**K_**m**_ (mM)**	**IC50**	**Substrate/ligand concentration (mM)**	**Target** **concentration** **(mM)**	**MIC (μM)^**b**^ (−P235) BL21**	**MIC (μM)^**b**^ (−P235) ATCC**	**MIC (μM)^**b**^ (+P235) BL21**	**MIC (μM)^**b**^ (+P235) ATCC**
P235	AP	277.2	315.8	6.30	10	20				
	SOD	12.45	42.3	4.37	2	4				
	GH17	0.003	0.042	1.95	1	25				
	LTP			1.43^a^	8	20	12.5	15	Growth	Growth
Effector 3	KTI			5.60^c^	4	10				
	ALDH	0.094	2.131	0.86	2	10				

*K_cat_ and K_m_ are given for the citrus AP, SOD, GH17, and ALDH. The IC_50_ (50% reduction in catalytic activity) by the effector is provided as the ratio of the substrate concentration*.

a *Lipid binding assay was performed for LTP to demonstrate the inhibitory effect of LasP_235_*.

b *Bactericidal effect of LTP was monitored using two E. coli strains: BL21 and ATCC25922; the corresponding MIC values are in μM. Addition of LasP_235_ at a concentration same as the MIC completely blocks the bactericidal effect of LTP on the two E. coli strains*.

c *Effector 3 is added in the Trypsin inhibition assay by KTI to determine the IC_50_*.

#### *In planta* Assays

**Figure 3:**
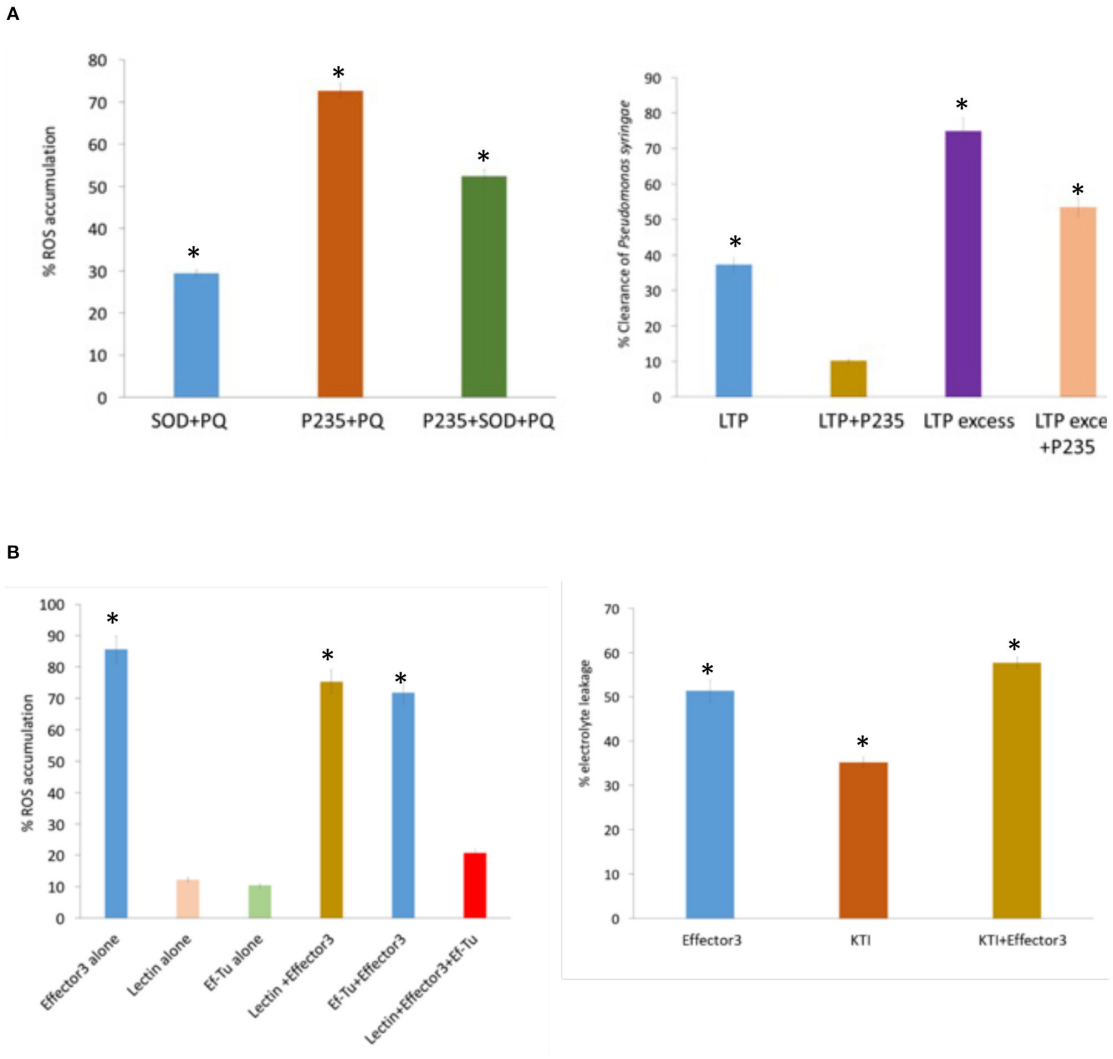
*In vitro* and *in planta* assays to demonstrate the inhibitory activity of the *C*Las effectors on their citrus protein targets. First, enzymatic, binding, or bactericidal assays are performed to determine the appropriate functional properties of the citrus target proteins and subsequently, the same assays are conducted to demonstrate the inhibitory activities of the *C*Las effectors on their citrus targets. [**(A)**, left] The inhibitory effect of *LasP*_*235*_ on SOD is monitored in tobacco by fluorescence microscopy. [**(A)**, right] Percentage reduction relative to the initial CFU of *E. coli* ATCC25922 by LTP alone and LTP plus *LasP*_*235*_. [**(B)**, left] The ability of Effector 3 to induce the ROS release in tobacco. The addition of lectin or Ef–Tu is not sufficient to suppress the ROS release by Effector 3. However, the combination of lectin and Ef–Tu significantly reduces the ROS release by Effector 3. [**(B)**, right] The electrolyte leakage due to ROS-produced by PQ is set 100%. A relative electrolyte leakage due to infiltration of Effector 3, KTI, and Effector 3 + KTI in tobacco. *Indicates statistical significance at *p* ≤ 0.05.

*In planta* assays for monitoring ROS production, bacterial clearance, and PCD induction were performed in tobacco to examine the inhibitory effect *LasP*_*235*_ and Effector 3 on their citrus target proteins. Paraquat was used for inducing the production of ROS in tobacco. The ROS level was monitored using a ROS/Superoxide detection assay (Zhou et al., 2018). In this experiment, the ROS level induced by agrobacterium carrying an empty vector (i.e., no gene) plus PQ was normalized to 100%. Note that, the infiltration of agrobacterium carrying citrus SOD reduced the ROS level significantly below 100%. However, as shown in Figure 3A (left), the simultaneous addition of agrobacteria carrying *LasP*_*235*_, and citrus SOD showed the elevation in the ROS level proving *in planta* inhibition of citrus SOD by *LasP*_*235*_. *In planta* bactericidal activity of citrus LTP was monitored by qPCR that showed the reduction of bacterial load in tobacco infected with *P. syringae* pv. DC3000. As shown in Figure 3A (right), agrobacterium carrying citrus LTP (0.4 × 10^8^ CFU ml^−1^) reduced the bacterial load to 37%. Increasing agrobacterium carrying citrus LTP by 10 times (i.e., 0.4 × 10^9^ CFU ml^−1^) led to the 75% reduction in the bacterial load. The addition of agrobacterium carrying *LasP*_*235*_ (0.4 × 10^8^ CFU ml^−1^) or 10 times of that increased the bacterial load. This proves that *LasP*_*235*_ is able to block *in planta* the bactericidal activity of the citrus LTP. Supplementary Table 2 shows the Ct values of a *Pst* gene (a measure of bacterial load) at different *LasP*_*235*_ concentrations. *In planta* studies were conducted in tobacco to examine the effect of (Effector 3–Lectin/Ef–Tu) interactions. As shown in Figure 3B (left), the infiltration of agrobacterium carrying Effector 3 induced ROS at a high level (85%). The ROS level due to agrobacterium carrying an empty vector plus PQ was set to 100%. Infiltration of agrobacterium carrying citrus Lectin or Ef–Tu had negligible effect on the ROS level. The co-infiltration of Effector 3 plus lectin or EF–Tu had very little effect on the ROS level induced by Effector 3 alone. However, combination of lectin and Ef–Tu was able to reduce the ROS level induced by Effector 3. In this regard, it is important to note that some bacteria, such as *Porphyromonas gingivalis, Mycobacterium tuberculosis, Helicobacter pylori*, and *Bacillus anthracis*, utilize ROS to support their growth and to establish infection (Paiva and Bozza, 2014) whereas plant lectin and Ef–Tu tend to inhibit ROS production or ROS-mediated signaling (Wang and Bouwmeester, 2017). It appears that pathogenic *C*Las may use Effector 3 to maintain ROS level that is beneficial to pathogen growth and infection by inhibiting anti-ROS citrus lectins and Ef–Tu. The PQ was also used to induce PCD *via* ROS in tobacco. The PCD was monitored by electrolyte leakage (Kacprzyk et al., 2016), which was set to 100% as induced by agrobacterium carrying an empty vector plus PQ. Infiltration of the agrobacterium carrying Effector 3 induced ~50% electrolyte leakage, which, as shown in Figure 3B (Right), was reduced on infiltration of agrobacterium carrying citrus KTI. The co-infiltration of Agrobacteria carrying citrus KTI and Effector 3 elevated PCD thereby confirming that Effector 3 is an inhibitor of the citrus KTI.

### To Predict and Validate the Molecular Mechanism of Effector–Target Inhibitory Interactions

**Figure 4:**
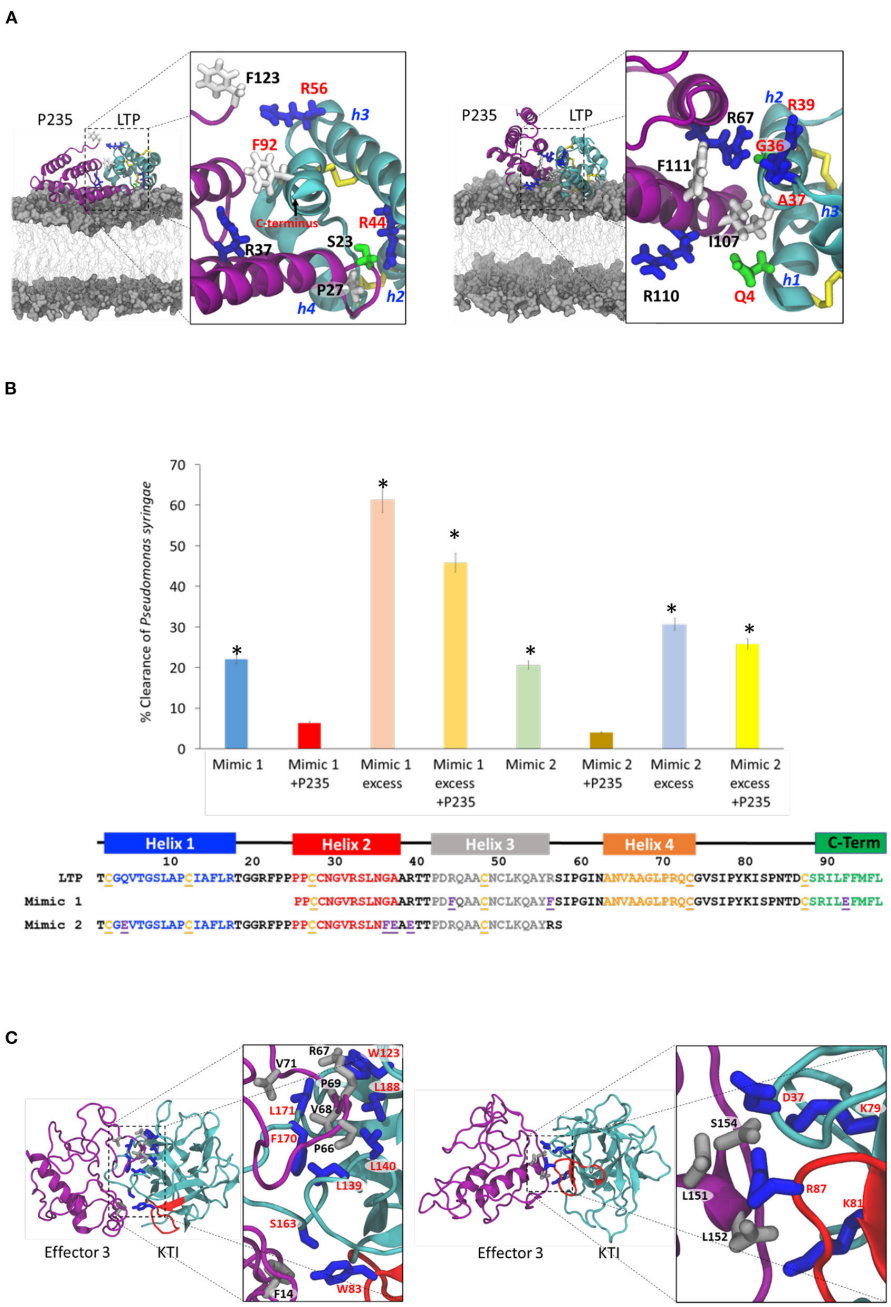
Prediction and validation of (citrus target–*C*Las effector) interaction at the contact interface. Molecular modeling is performed to predict the pairwise interactions based on which mimics are designed to displace the effector from the (citrus target-*C*Las effector) complex. Finally, experiments are performed to determine if the mimic, indeed, displaces the effector from the complex and if so, our prediction of the pairwise interactions are validated. **(A)** Two possible modes of interactions between *LasP*_*235*_ (magenta ribbon) and LTP (cyan ribbon) which block the membrane attachment of LTP thereby inhibiting the bactericidal activity. The disulfide bridges are shown as yellow sticks. The LTP helices (h2, h3, and h4) and the C-terminal segment are predicted to be involved in one mode of interaction. The LTP helices (h1, h2, and h3) are involved in the other mode of interaction. Important residues in the pairwise contacts are shown: *LasP*_*235*_ residues are labeled black whereas the LTP residues are labeled red. Basic, acidic, neutral, and acidic residues are, respectively, as blue, red, green, and gray sticks. **(B)** Amino acid sequences of Mimic 1 and Mimic 2 at the bottom. Bacterial (*P. syringae* pv. tobaci) clearance in tobacco by the two mimics in the presence and absence of *LasP*_*235*_. Note that an excess of the mimics is needed for significant bacterial clearance. Mimic 1 is a better bactericidal than Mimic 2. *LasP*_*235*_ is an inhibitor of Mimic 1 or Mimic 2. **(C)** Two models of interactions between Effector 3 (magenta ribbon) and KTI (cyan ribbon with the reactive loop in red). Both models show interactions with the KTI reactive loop as a prominent mode of inhibition. The predicted pairwise interactions are shown. The residues from Effector 3 are shown as gray sticks and labeled black whereas the residues from KTI are shown as blue sticks and label red. *Indicates statistical significance at *p* ≤ 0.05.

We performed all-atom MD simulations (Li et al., 2018) to predict the interactions that stabilize the (inhibitory *CLas* effector–citrus protein target) complexes. Initially, we focused on the bactericidal effect of LTP. As described in Materials and Methods section, we first obtained an optimized homology-based model of the citrus LTP as shown in Supplementary Figure 1C. Then, we performed MD simulations in (water: lipid) bilayer for 10 μs. As described in the Supplementary Figure 4A, MD simulations revealed that the LTP helices h2, h3, h4, and the C-terminal loop were involved in interaction with the lipid bilayer defining membrane attachment, which is the first step in the bactericidal activity. The interactions of the positively charge arginine residues R21, R32, R39, R44, R71, and R89 (shown in blue in Figure 4B) with the negatively charged lipid polar heads appear to be extremely critical for the LTP membrane attachment. To study the interaction of *LasP*_*235*_ with LTP, we docked the homology based *LasP*_23**5**_ model to the optimized LTP model. We then performed MD simulations of the LTP-*LasP*_*235*_ complex in aqueous environment for 6 μS to determine which mode of *LasP*_**235**_ binding may block the LTP attachment to the lipid bilayer as discussed in Supplementary Figures 4A,B. One mode of *LasP*_*235*_ (magenta) interaction, shown in Figure 4A (left), involves the LTP (cyan) helices h2, h3, and h4 and the C-terminal loop resulting in partial blocking of the B1 LTP site by *LasP*_*235*_. The prominent pair-wise contacts between *LasP*_*235*_ (magenta) and LTP (cyan) are predicted from MD simulations using the method described in Supplementary Figure 4. They are as follows: S23-R44, P27-R44, R37-F92, and F123-R56. Another mode of *LasP*_*235*_ binding as shown in Figure 4A (right) involves the LTP helices h1, h2, and h3 with pairwise contacts: I107-R39, R110-A37, R110-Q4, and F111-G36. In both binding modes, the LTP attachment to the bacterial membrane is partially blocked. Based on the two modes of interactions, we designed two LTP mimics shown in Figure 4B, i.e., Mimic 1 containing h2, h3, h4, and the C-terminal loop and Mimic 2 containing h1, h2, and h3. We also introduced amino acid substitutions, i.e., R44F, R56F, and F92E in Mimic 1 and Q4E, G36F, A37E, R39E in Mimic 2. These amino acid substitutions are predicted to increase the strength of pairwise interactions between LTP and *LasP*_*235*_ as listed above. While both mimics are predicted to partially block the inhibitory activity of *LasP*_*235*_ on bactericidal LTP, Mimic 1 is supposed be a better blocker than Mimic 2. Our predictions are validated by the results of *in planta* tobacco studies shown in Figure 4B. Here, Mimics 1 and 2 were infiltrated to express at the same and 10 times level of *LasP*_*235*_. The results show that: (i) both mimics by themselves show bactericidal effect on *P. syringae* pv. tobaci but smaller than the full-length LTP; and (ii) Mimic 1 is better *LasP*_*235*_ blocker/bactericidal than Mimic 2. These experimental observations are in full agreement with our predictions. Therefore, we may conclude that interactions shown in Figure 4B (left) is the most prominent mode of LTP blocking by *LasP*_*235*_. Supplementary Figure 4B shows the Ct values of a *Pst* gene (a measure of bacterial load) due to treatment of Mimics 1 and 2 at different concentrations.

We constructed two models of (Effector 3: KTI) complex with Effector 3 and Kunitz represented respectively by purple and cyan ribbons. Both complexes are chosen to block the reactive KTI loop (residues 82–94) as shown in the homology-based model of Supplementary Figure 1D. Blocking of the KTI reactive loop is critical in trypsin protease inhibition. We performed 2 μS MD simulations on these two complexes in aqueous environment. Figure 4C shows two different ways Effector 3 may block the KTI reactive loop (shown in red). The sampling of the MD trajectories reveals the following dominant pairwise interactions with C^α^-C^α^ distance <4Å as described in Supplementary Figure 5. Stabilizing pairwise interactions in one model in Figure 4C (left) are as follows: F14-W83, P69-W123, V68-L139, V68-L140, F14-S163, V68-F170, L71-L171, and P69-L188 whereas in the other model in Figure 4C (right), and they are as follows: L151-D37, L151-R87, L152-K81, and S154-K79. In these pairwise interactions, Effector 3 and KTI are shown, respectively, as gray and blue ball-and-stick representations. The mutational studies are needed to discriminate the two modes of inhibition of KTI by Effector 3 described in Figure 4C.

## Discussion

**Figure 5:**
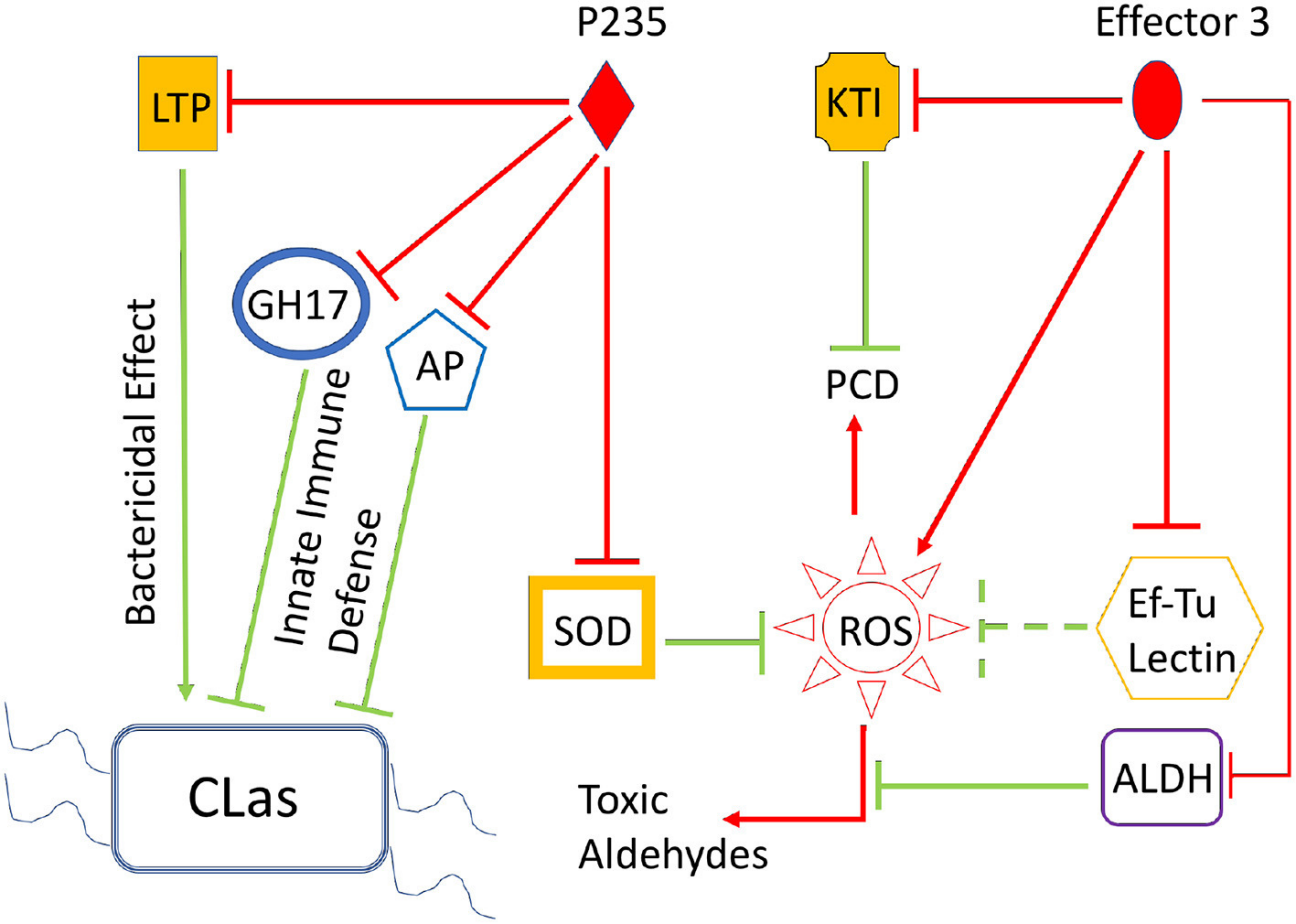
Combined effect of the *C*Las effectors, *LasP*_*235*_ and Effector 3, on the citrus innate immune due to the target proteins. The *LasP*_*235*_ targets shown here are SOD, LTP, KP, and GH17 whereas the Effector 3 targets are as follows: ALDH, Lectin, KTI, and Ef–Tu. All these citrus targets participate in innate immune defense (shown as green arrows) during bacterial infection. For example, SOD controls the level of ROS such that the beneficial effects of ROS-induced immune defense can be harnessed without the level of ROS level exceeding a critical threshold over which there may cause host damage. EF–Tu and ALDH also limit the level of ROS. The GH17 and KP offer immune defense against bacterial infection. LTP can directly exert bacterial effect whereas KTI prevents premature PCD, which may help bacterial growth and infection. The effects of pathogenic *C*Las proteins, *LasP*_*235*_ and Effector 3, are shown as red line arrows (promoting a process and red line blockers (as inhibiting a process). The combined effects of *LasP*_*235*_ and Effector 3: elevation of ROS level, premature PCD, and inhibition of *C*Las clearance.

Bacterial effectors are known to inhibit plant innate immune signaling networks mediated by PTI, ETI, and plant SA, JA, and ET hormones. The end products of PTI, ETI, and plant hormone signaling are the PR or immune defense proteins that either clear the invading the pathogen or block infection. Typically, each immune defense protein is induced at a low level and a single protein; therefore, can neither completely clear the pathogen nor can it totally block the infection. Interestingly, simultaneous induction of multiple immune defense proteins (albeit at low levels) can lead to effective clearance of the invading bacteria and blocking of infection caused by them. However, the multiple effectors from a pathogenic bacterium such as *C*Las can suppress multiple signaling steps to support bacterial growth and infection. Here, we report the role of two *C*Las effectors, *LasP*_*235*_ and Effector 3, in HLB pathogenesis. The each of them may directly target and inhibit more than one citrus innate immune defense proteins involved in bactericidal and/or disease-blocking activity. For example, *LasP*_*235*_ can inhibit the citrus targets (SOD, AP, GH17, and LTP) whereas Effector 3 can inhibit citrus targets (KTI, ALDH, Lectin, and Ef–Tu). Although, as shown here, a bacterial effector may target several plant proteins, inhibitions of all targets may not be equally important for bacterial pathogenesis. A direct evaluation of the importance of each (plant protein–bacterial effector) interaction is traditionally obtained by knockout of a specific bacterial effector. Since *C*Las is not culturable, it is not possible to conduct gene knockout experiments. However, the inhibitory activities of a *C*Las effector against different citrus targets reveal qualitatively the relative importance of different inhibitory (*C*Las effector–citrus target) interactions in HLB pathogenesis. For example, as shown in Table 1, *LasP*_*235*_ is a potent inhibitor of LTP because at equimolar concentration it can completely block the bactericidal activity of LTP. Thus, *LasP*_*235*_ may play an important role in HLB pathogenesis. Note that, relatively low IC_50_ values (within 1 to 6) in Table 1, argue that the corresponding inhibitory interactions may be relevant in HLB pathogenesis. Figure 5 schematically summarizes the combined effect of the inhibitory interactions of *LasP*_*235*_ and Effector 3 on their citrus targets as determined from our *in vitro* and *in planta* studies. The immune stimulatory defenses exerted by the identified citrus targets are marked by green lines whereas the inhibition of these targets by the two effectors *LasP*_*235*_ and Effector 3 of pathogenic *C*Las are marked by red lines. Note that SOD reduces the level of ROS whereas Ef–Tu, Lectin, and ALDH tend to control the toxic damage due to ROS. The GH17 and AP provide immune defense *via* SA-signaling, which may involve ROS production whereas KTI may prevent premature ROS-induced PCD and *LasP*_*235*_ may block *C*Las clearance by LTP. Thus, *LasP*_*235*_ and Effector 3, target and interact with the ROS, PCD, and bactericidal pathways in a way that adversely affect citrus innate immune defense and in turn, facilitate HLB pathogenesis.

We analyzed the detailed interactions at the contact interfaces of the (*LasP*_*235*_-LTP) and (Effector 3-KTI) complexes. The molecular modeling and mutational analysis revealed the predominant mechanism of LTP inhibition by *LasP*_*235*_. We were able to design Mimic 1 (derived from LTP with specific amino acid substitutions) that showed intrinsic bactericidal activity and exhibited *LasP*_*235*_ inhibitory activity. The Mimic 1 can be further modified to increase its *LasP*_*235*_ inhibitory and bactericidal activity. We have also obtained two modes of inhibition in which Effector 3 may block the reactive loop of the citrus KTI. We have not yet completed *in plant*a experiments to determine whether one of the two modes of inhibition or both may be important. Nonetheless, the citrus KTI as the target of inhibition by a *C*Las effector is an interesting observation since such inhibition may cause premature PCD, which may be beneficial to *C*Las in causing infection (Randow et al., 2013).

## Data Availability Statement

The raw data supporting the conclusions of this article will be made available by the authors, without undue reservation.

## Author Contributions

GG contributed to project planning, experimental work, data analysis, and writing of the manuscript. SB performed the majority of *in vitro* and *in planta* studies, data analysis, and writing of the manuscript. LH performed the MD simulations and analyses. HN assisted in the split GFP assays. RR performed expression of some recombinant proteins. JV performed some MIC assays. ES supervised the work of GM and QS on citrus infection by *C*Las and protein extraction from healthy and infected citrus and the work of GH and SZ toward generating transgenic tobacco expressing GFP1-9. All authors contributed to the article and approved the submitted version.

## Funding

This work was funded by the NIFA-CDRE grant (Fed Award # 2018-70016-27453 to PI: Jinhe Bai, USDA-ARS; Co-PI: Subaward # 59-6034-8-005) on accelerating implementation of HLB hybrids as new commercial cultivars for fresh and processed citrus through a collaboration between Gupta (New Mexico Consortium) and Stover (USDA-ARS) labs.

## Conflict of Interest

The authors declare that the research was conducted in the absence of any commercial or financial relationships that could be construed as a potential conflict of interest.

## Publisher's Note

All claims expressed in this article are solely those of the authors and do not necessarily represent those of their affiliated organizations, or those of the publisher, the editors and the reviewers. Any product that may be evaluated in this article, or claim that may be made by its manufacturer, is not guaranteed or endorsed by the publisher.
