# Supplementary material for: Two Liberibacter Proteins Combine to Suppress Critical Innate Immune Defenses in Citrus
Source: Front Plant Sci. 2022 May 2;13:869178. doi: 10.3389/fpls.2022.869178 (PMC9108871; doi:10.3389/fpls.2022.869178)

**SUPPLEMENTARY INFORMATION**

**Two Liberibacter proteins Combine to Suppress Critical Innate Immune Defenses and Facilitate Huanglongbing Pathogenesis in Citrus**

Supratim Basu^$^, Loan Huynh^$^, Shujian Zhang^$^, Roel Rabara^$^, Hau Nguyen^$^, Jeanette Valesquez^$^, Guixia Hao^#^, Godfrey Miles^#^, Qingchun Shi^#^, Ed Stover^#^, and Goutam Gupta^$*^

^$^Biolab, New Mexico Consortium, 100 Entrada Drive, Los Alamos, NM 87544, USA

^#^U. S. Horticultural Research Laboratory 2001 South Rock Road Fort Pierce, FL 34945, USA

*Correspondence: ggupta@newmexicoconsortium.org

**Fig. S1.** Homology based and energy-minimized models of **(A)** *Las_P235_*, **(B)** Effector 3, **(C)** LTP, and **(D)** KTI, the reactive loop of which is shown as space-filling representation.

**Fig. S2.** Western blot analysis of the citrus targets for **(A)** *LasP_235_* and **(B)** Effector 3 by an anti-His_6_ antibody. In (A) Lanes, 1: Marker; 2: Aspartyl Protease; 3: Glycosyl Hydrolase 4: Superoxide Dismutase; 5: Lipid Transfer Protein. In (B) Lanes, 1: Marker; 2: PSII subunit protein; 3: Aldehyde dehydrogenase; 4: Kunitz trypsin inhibitor; 5: Lectin like protein.

**Fig. S3**. **(A)** Schematic representation predicted models of interaction between LTP and lipid bilayer. Grey lines and surfaces represent lipid acyl chains and head groups, respectively. LTP is shown as a cyan ribbon. LTP-bilayer interaction involves LTP helices h2, h3, h4 with C-terminus segment inserting into the bilayer. Our MD simulations suggest that positively charged residues R21, R32, R39, R44, R71 and R89 (blue sticks) are critical for the interaction with the lipid bilayer. One of LTP lipid entrance sites (B1) is formed by C-terminus and the beginning of h3 with R44 interacting with bilayer membrane. The other LTP lipid entrance site (B2) is formed by C-terminus and loop connecting h3 and h4 and are solvated by water. Other residues at the LTP-lipid interface and water are not shown. Total simulation time was 1-ms. Disulfide bridges for the pairs C2-C50, C12-C27, C28-C73, and C48-C87 are represented with yellow sticks. Amino acid sequence for LTP with residues involved in disulfide bond are underlined: TCGQVTGSLA PCIAFLRTGG RFPPPPCCNG VRSLNGAART TPDRQAACNC LKQAYRSIPG INANVAAGLP RQCGVSIPYK ISPNTDCSRI LFFMFL. **(B)** Heatmaps depict residue-specific distributions of the distance between each C_α_ atom and the bilayer center along its normal of LTP-membrane separation. Two independent molecular simulations of LTP- membrane (top and bottom) were conducted with random initial location of the LTP in water. Dashed red line at 2.0 nm indicates the average position of lipid phosphorus atoms. Residues that from disulfide bridge are in yellow (C2-C50, C12-C27, C28-C73, and C48-C87). Both (top and bottom) MD simulations show part of helix 4 and C-terminus of LTP insert deeper into the bilayer, with helix 2 and the beginning of helix 3 interacting with lipid head groups. As shown, one of LTP lipid entrance sites is formed by C-terminus and the beginning of h3 with R44 interact with bilayer membrane. The other LTP lipid entrance site is formed by C-terminus and loop connecting h3 and h4. The C-terminal residues are either in the disordered loop or helical conformation. The total simulation time was 10 ms per system.

**Fig. S4**. Heatmaps depict pairwise residues interaction between C_α_ atoms of LASP235 and LTP with C_α_-C_α_ interaction distance ≤ 4.0 Å. MD simulation of LASP235-LTP were conducted in the presence of lipid bilayer. LASP235 either interacts with **(A)** helix 2, 3, 4 and the C-terminal segment with or **(B)** helix 1, 2, and 3 of LTP.

**Fig. S5**. Heatmaps depict pairwise interactions between C_α_ atoms of Effector 3 and KTI residues with C_α_-C_α_ interaction distance ≤ 4.0 Å. Active loop of KTI is comprised of residues 82 to 94. Signaling sequence of KTI (residues 1-22) was not included in the simulation model. MD simulations of the (Effector 3-KTI) complex were conducted in the presence of water. Two modes of interaction were predicted: **(A)** minimal interactions of Effector 3 with the KTI active loop residues and **(B)** direct interactions of Effector 3 with the KTI active loop residues, R87 of KTI.

**Table S1.** An expanded list of putative citrus targets of **(A)** *C*Las-LASP235, **(B)** *C*Las-Effector 3, and (C) control buffer obtained following the method described in Fig. 1 in the main text.

**Table S2.** Ct values obtained from qPCR analysis after infiltration of *N.benthamiana* leaves with (A) For LTP +LASP235 and (B) LTP +LASP235+ Mimic1 and M2.

**Figure S1**

**Figure S2**

**
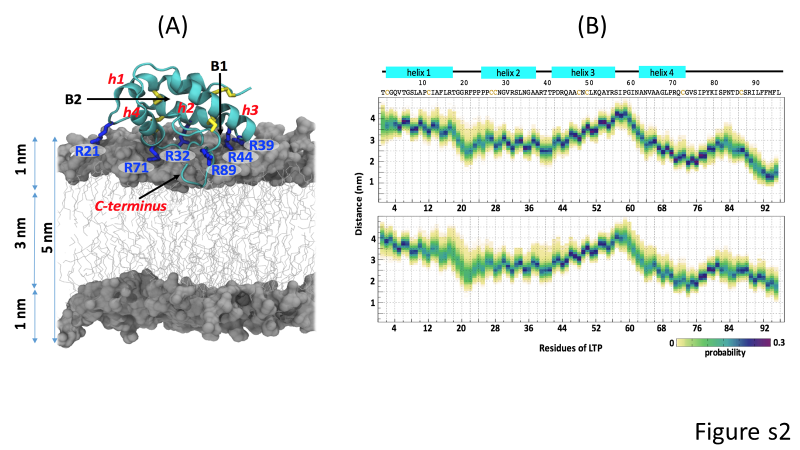
Figure S3**

**Figure S4**

**Figure S5**


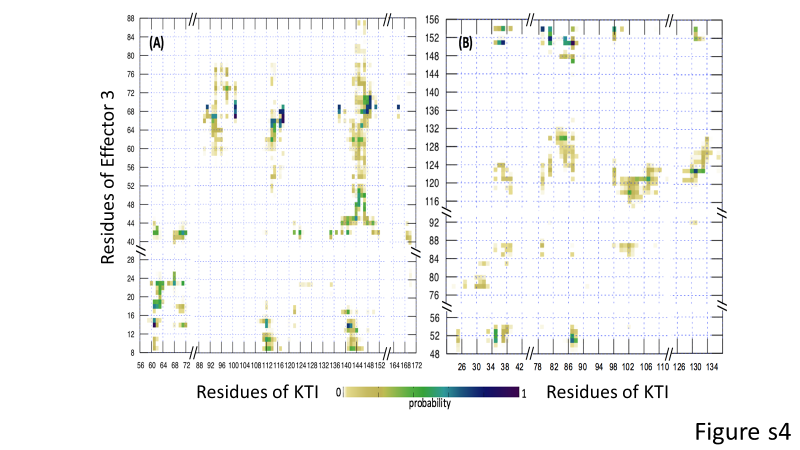


**Table S1A**


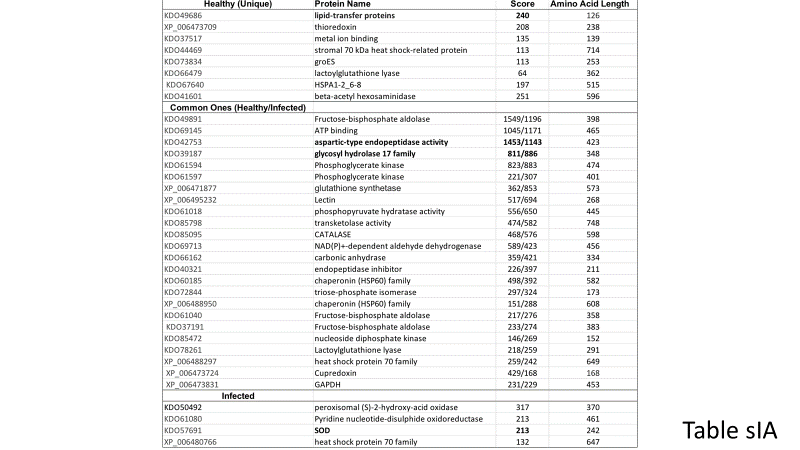


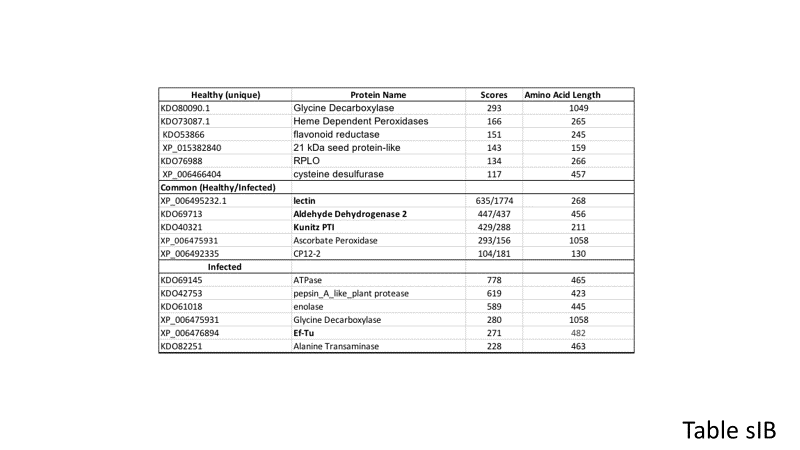
**Table S1B**

Table S1C


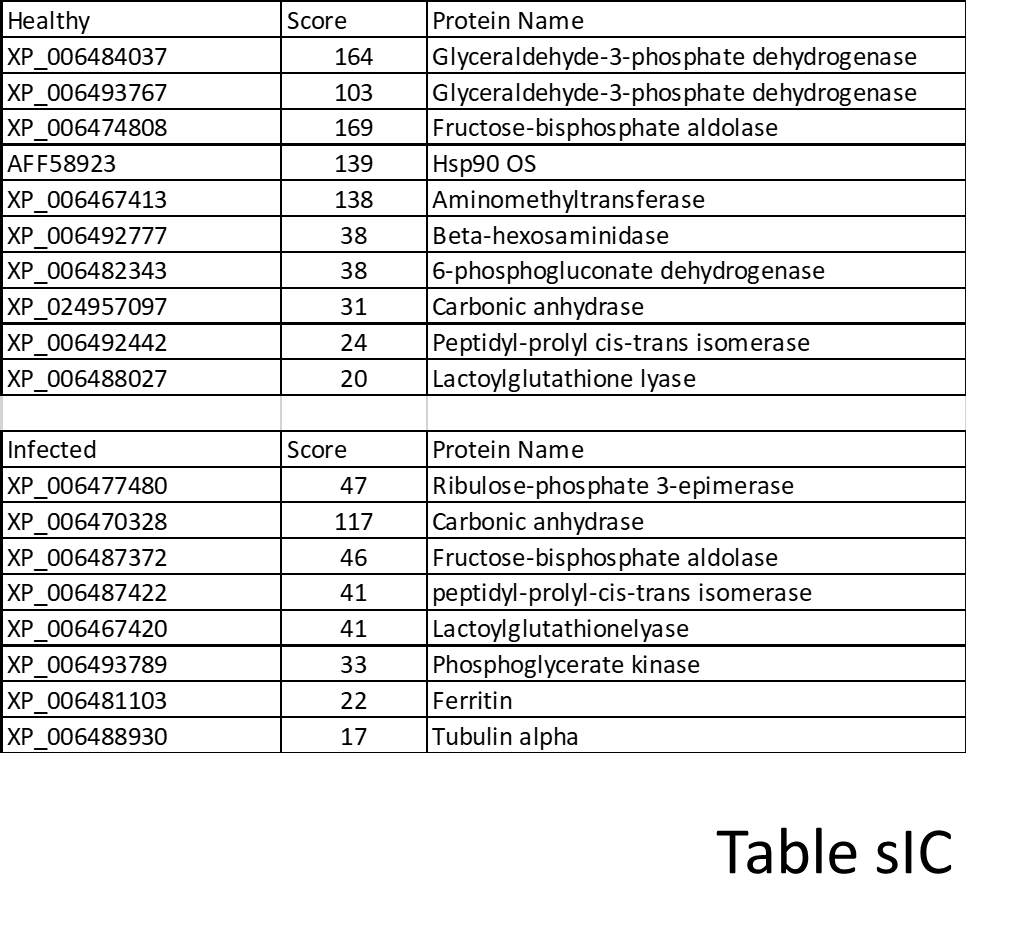


**Table S2**


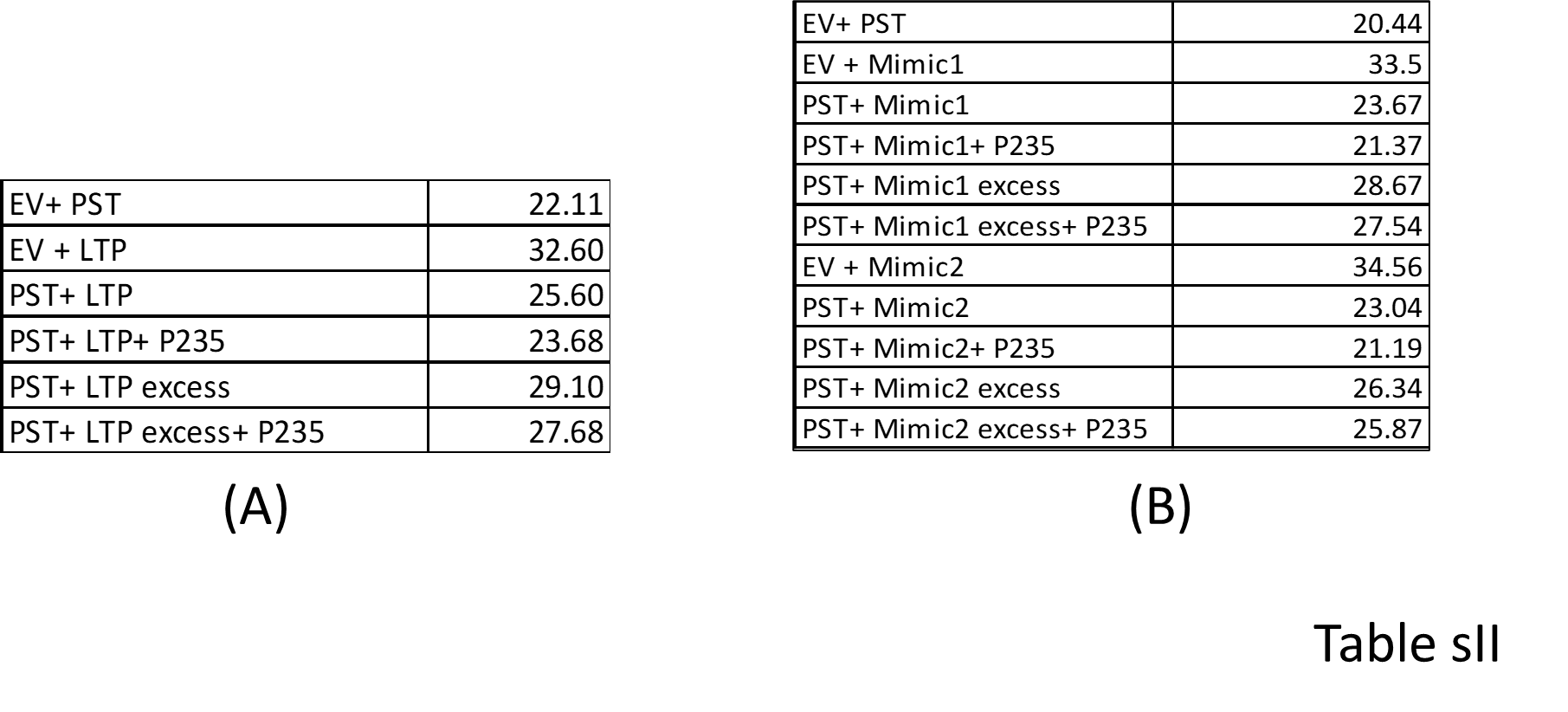

Supplement: Supplementary file 1 [file Data_Sheet_1.docx]
